# Analyzing the demographic, spatial, and temporal factors influencing social contact patterns in U.S. and implications for infectious disease spread

**DOI:** 10.1186/s12879-021-06610-w

**Published:** 2021-09-27

**Authors:** Audrey M. Dorélien, Aparna Ramen, Isabella Swanson, Rachelle Hill

**Affiliations:** 1 University of Minnesota, Minneapolis, MN USA; 2 US Census Bureau, Suitland, MD USA

## Abstract

**Background:**

Diseases such as COVID-19 are spread through social contact. Reducing social contacts is required to stop disease spread in pandemics for which vaccines have not yet been developed. However, existing data on social contact patterns in the United States (U.S.) is limited.

**Method:**

We use American Time Use Survey data from 2003–2018 to describe and quantify the age-pattern of disease-relevant social contacts. For within-household contacts, we construct age-structured contact duration matrices (who spends time with whom, by age). For both within-household and non-household contacts, we also estimate the mean number and duration of contact by location. We estimate and test for differences in the age-pattern of social contacts based on demographic, temporal, and spatial characteristics.

**Results:**

The mean number and duration of social contacts vary by age. The biggest gender differences in the age-pattern of social contacts are at home and at work; the former appears to be driven by caretaking responsibilities.

Non-Hispanic Blacks have a shorter duration of contact and fewer social contacts than non-Hispanic Whites. This difference is largely driven by fewer and shorter contacts at home. Pre-pandemic, non-Hispanic Blacks have shorter durations of work contacts. Their jobs are more likely to require close physical proximity, so their contacts are riskier than those of non-Hispanic Whites. Hispanics have the highest number of household contacts and are also more likely to work in jobs requiring close physical proximity than non-Hispanic Whites.

With the exceptions of work and school contacts, the duration of social contact is higher on weekends than on weekdays. Seasonal differences in the total duration of social contacts are driven by school-aged respondents who have significantly shorter contacts during the summer months. Contact patterns did not differ by metro status. Age patterns of social contacts were similar across regions.

**Conclusion:**

Social contact patterns differ by age**,** race and ethnicity, and gender. Other factors besides contact patterns may be driving seasonal variation in disease incidence if school-aged individuals are not an important source of transmission. Pre-pandemic, there were no spatial differences in social contacts, but this finding has likely changed during the pandemic.

**Supplementary Information:**

The online version contains supplementary material available at 10.1186/s12879-021-06610-w.

## Introduction

Emerging infectious diseases such as SARS-CoV-2, which causes coronavirus disease 2019 (COVID-19), pose a substantial challenge to global and US public health. SARS-CoV-2, a respiratory pathogen, spreads primarily through direct in-person social contacts [27] and time spent in locations such as schools and the workplace greatly influences the number and duration of these contacts [10]. The challenge of effectively responding to respiratory pathogens is greater when treatments and vaccines are not yet available. Therefore, in the period between the onset of a pandemic and the development of treatments and creation of a vaccine, stopping the spread of infectious disease becomes a question of promoting non-pharmaceutical interventions (NPIs) [43]. NPIs such as school closures and social distancing measures require most individuals (those not classified as essential workers) to stay at home except for taking essential trips to get food or medicine. Using such interventions correctly requires a better understanding of social contact patterns, which are a critical factor in the transmission and control of infectious diseases such as coronavirus and influenza [25].

Social contact patterns vary by population, so context-specific estimates are necessary to tailor interventions to the country or region of interest [34]. Unfortunately, there is a paucity of empirical data on social contacts from the US [8, 21]. As a result, current US interventions are often difficult to target, leading to suboptimal outcomes. Moreover, when capacity for testing is limited early in an epidemic, as with COVID-19, it can be targeted towards regions, populations, and settings most likely to have high community spread when social contact patterns are known, facilitating identification of both mildly symptomatic and asymptomatic carriers and preventing transmission by super-spreaders. When vaccines become available, a lack of accurate information impacts optimal vaccine distribution as well as vaccination booster schedules [14]. In order to fill this information gap, we describe and quantify US social contact patterns using data from the Bureau of Labor Statistics’ American Time Use Survey (ATUS), 2003–2018.

### There are five different approaches for estimating age contact patterns

Social contact patterns can be summarized with age-contact matrices [44]. Typically, cells in the age-contact matrices display the average number of daily contacts (e.g., close proximity conversation and/or physical contact) that respondents in one age group have with individuals in another age group. These matrices can capture many of the important components of social contact structures such as typical household composition, daily routines, and activities (e.g., school and work), and can be used as inputs into infectious disease models.

In particular, five main approaches to measure contacts directly from social data have been proposed [24, 44, 46]. The first and most common approach relies on contact surveys in which the respondent self-reports the number of contacts they encountered during a randomly sampled day [3, 11, 34, 44]. Additional information captured in the survey includes the age/sex of contacted persons, type of contact, duration, location, and frequency of contacts. The best-known study to use this approach is the Mossong et al. [34] study, which collected contact information from 7,290 participants in 2006 from eight different European countries as part of the POLYMOD (Improving Public Health Policy in Europe through Modelling and Economic Evaluation of Interventions for the Control of Infectious Diseases) project funded by the European Commission. They recorded contacts over a 24 h period using paper diaries in which information on the demographics of contacted persons, the location, frequency, duration, and type of contact (physical or non-physical) were collected. They found that age-specific social contact patterns do vary by country and that the differences are epidemiologically meaningful. Over the past ten years there have been additional POLYMOD-like studies conducted in Vietnam, Zimbabwe, and Russia, as well as a few other developed countries [1, 21, 23, 32]. More recently, Klepac et al. [29] modernized this approach by combining a cellphone app (which records location each hour over a 24 h period) with self-reported contact data recorded at the end of that 24 h period. No such equivalent study exists for the US as a whole. Thus many researchers have used the POLYMOD data from UK and Germany, which are already more than 10 years old, as a substitute for US data [12, 31].

In a second approach, contact matrices are estimated from simulated output of individual-based models, appropriately calibrated to socio-demographic and time-use data, to generate the underlying contact network structure of the population [6, 24].

The most recently developed and third approach creates a model that simulates individual-level contacts based on POLYMOD data but uses different inputs from surveys such as the Demographic and Health Surveys (DHS) and International Labor Organization. This approach lacks individual-level contact data, but includes data on household age structure, population age composition, labor force participation, and other factors that strongly influence individual-level contact patterns [36], [33].

The fourth approach relies on time-use data and generates “time-of-exposure” age matrices (matrices of "who spends time with whom") by age. The ages of the respondents’ contacts are generated by assuming that for single activity/locations and relatively small time intervals, people mix with each other proportionally to the relative presence of their age group in the location [46].

Wireless wearable sensors such as motes, which regularly record the distance and duration of contact with other sensors within three meters, represent a fifth approach that can be used to measure contacts [20, 38]. This approach allows researchers to capture networks and is not subject to the under-reporting of brief contacts that can occur from self-reported data collection [39]. A big limitation is that these devices are typically deployed in small locations with bounded populations such as schools or hospitals since both members of each contact pair must have a sensor; therefore, these sensors cannot capture contacts at larger spatial scales. Identifying and personal information (such as name, grade level, etc.) about contacts is typically extracted from motes and analyzed. However, because motes are used in small, closed systems with a high degree of adoption, they are more useful for network analysis and evaluation of connectivity and clustering than mobile phone data [20].

Similar technology to motes is also found in many mobile phones, and as a result of the COVID-19 pandemic, a plethora of contact tracing apps have been developed. These apps use the smartphone’s Bluetooth signal to detect other devices in close proximity and then swap randomly generated anonymous ID codes. If a user tests positive, they can use the app to notify other users that they have been exposed. These apps have not been adopted by a large fraction of the population, so some contacts will be missed [30]. Due to mobile data privacy laws and concerns, these apps do not collect or share personally identifying information such as age with each other or government health agencies, which limits their application for research. However, they are useful in reducing transmission, especially for non-household contacts [45].

Mobile data, aggregated and anonymized by mobile networks, may also be useful for models of disease transmission between regions. They can reveal large-scale patterns of activity, population densities, and travel between regions [16]. They have been used to generate mixing matrices by region in COVID-19 transmission models [48]. However, mobility patterns did not appear to be strongly associated with COVID-19 transmission beyond the initial phase of the pandemic, and parameterizing transmission models using mobility data alone is likely to result in poorly performing models [2]. Moreover, it is difficult to create age-structured contact matrices from aggregated call records and mobility data [35]. Mobile network and app data will also have selection bias and limited generalizability to the overall population due to age, sociodemographic, and geographic differences in mobile phone ownership and app usage. Without being coupled to a survey or widely used digital contact tracing apps that collect and share age, which is unlikely with current mobile data privacy laws and concerns, mobile data cannot tell us which age groups are interacting with each other, nor the duration and nature of those contacts.

### Existing information on US social contact patterns

As mentioned above, relatively little is known about US contact patterns. What is known has either been based on geographically small populations, which may not be generalizable [7] or does not describe variation in social contacts across time and space [46]. DeStefano et al. [7] conducted a study of social contact patterns in four small North Carolina counties during the 2007–08 influenza season and found that the number of contacts varied with age and was lower on weekends than weekdays. They also found that for adults, the number of contacts increased during times of peak influenza activity but that this was not the case for children. There was also evidence of seasonal variation in mean daily contacts, but since this data was limited to one year they could not be certain if the pattern repeats every year [7].

Since there are no national US surveys of contact structures, Zagheni et al. [46] used a single year of ATUS data to summarize one aspect of contact patterns—the duration of time people spend with other people of different ages. They found that people tend to spend more time with individuals of the same age and with individuals one generation apart, such as parents interacting with children and vice versa. They illustrated that a model of age-specific immunity to varicella that incorporated the contact matrices from the time-use survey was able to predict US varicella seroprevalence well. Zagheni et al.’s [46] results have three important limitations that are addressed by our study. First, Zagheni et al. are unable to study how contact patterns may have changed over time because they only use a single year of ATUS data. Second, they do not examine seasonal variations in contact patterns, which are known to be important drivers of the spread of close-contact diseases. Third, they do not examine spatial variation in contact patterns across the US. We are working with and building on this earlier work by taking advantage of the multiple years of data now available in the ATUS to identify meaningful sources of variation in contact patterns over time and across seasons and space.

### Paper contributions

Time-use diaries such as the ATUS contain some of the same information that is present in social contact surveys. Specifically, the number and duration of contacts in the respondents’ households as well as the age and sex of the respondents’ household members. Therefore, in this paper, we use the social contact survey approach to generate empirically based estimates of the age pattern of **mean duration** (in minutes), **mean number** of contacts, as well as **age contact matrices** (who spends time with whom by age) for household contacts in the US. We also estimate the age pattern of the number and duration of social contacts for other locations (all locations, home/yard, work, public location), but we are not able to generate age contact matrices directly from the data. In future work, we will use proportionate time mixing assumptions to create age-specific contact matrices (showing duration of contacts) for non-household locations.

Because we have a large dataset that spans the years 2003–2018, **we can describe and test for differences in these social contact patterns by respondent’s age group, gender, and ethnicity/race. We also describe contact patterns across different spatial (e.g., metro vs non-metro, regions) and temporal scales (e.g., day of the week, seasons)**. We hope that this information can be used to parameterize models for the spread of close-contact infectious diseases, such as COVID-19, influenza, and measles, while helping to identify groups and settings to target for testing and interventions. The information generated in this paper can also be used to evaluate the effectiveness of social distancing measures as a result of the COVID-19 pandemic [26]. Future work will identify the main social and demographic determinants of US contact patterns.

## Data and methods

### ATUS data

The Bureau of Labor Statistics’ American Time Use Survey (ATUS) has been fielded continuously from 2003 to present and focuses on time use in the United States. The goal of the survey is to measure how people divide their time among the various activities of daily life. Survey participants are asked to recall all the activities that took place in the 24 h of the day preceding the survey. Data are collected in the form of diaries in which respondents describe their daily activities chronologically in increments as small as one minute, and are publicly available. Diaries cover all seasons, days of the week, and holidays.

The ATUS sample is drawn from past participating households of the Current Population Survey (CPS). Similar to the CPS, ATUS does not include active military personnel and people residing in institutions such as nursing homes and prisons. The ATUS sampling strategy is a three-stage design that stratifies the sample by state and demographic characteristics (including race and ethnicity, age and presence of children in the household, and number of adults in adults-only households); within households, individuals over the age of 15 are then sampled at random. Finally, respondents are asked about one diary day. Weekend days are oversampled (Table 1) but within the weekend and weekday categories, days of the week are randomly surveyed. We employ weights (wt06) to adjust for oversampling by race/ethnicity, presence of children, and day of the week as well as differential response rates. Including weights in our analyses leaves us with a sample that is representative of all residents living in households in the United States who are at least 15 years old (see weighted sample compositions included in Table 1). Additional information about the survey’s sample design can be found in the American Time Use Survey User’s Guide – Understanding the ATUS 2003-2018 [42].

**Table 1:** Counts and weighted proportions illustrating some of the sociodemographic, spatial, and temporal variation available in the ATUS data set. Sample excludes Alaska and Hawaii

Total Sample N = 200,136 (excluding AK & HI)							
**Sex**	**N (wgt %)**		**Presence of kids in HH**			**Race/Ethnicity**	
Male	87,850 (48.4)		No	109,616 (59.8)		Non-Hispanic White	136,252 (68.2)
Female	112,286 (51.6)		Yes	90,520 (40.2)		Hispanic	27,273 (14.7)
						Non-Hispanic Black	26,775 (11.7)
						Non-Hispanic Other	9,836 (5.4)
**Economy**			**Type of day**			**Employment status**	
Recession (12/01/2007–06/30/2009)	20,464 (9.7)		Weekday	99,711 (71.4)		Employed	124,356 (62.9)
			Weekend	100,425 (28.6)		Not in the labor force	66,503 (5.4)
Non recession	179,672 (90.3)					Unemployed	9,277 (31.7)
**Education status**			**Season**			**Age**	
Less than high school	30,801 (17.3)		Winter	51,874 (25.0)		15–19	12,173 (8.6)
High school degree	52,038 (29.3)		Spring	50,621 (25.0)		20–24	8,734 (8.6)
Some college	53,695 (25.0)		Summer	49,276 (25.0)		25–29	13,748 (8.6)
Bachelors degree	39,692 (18.1)		Fall	48,365 (25.0)		30–34	18,459 (8.3)
Advanced degree	23,910 (10.2)					35–39	20,225 (8.3)
						40–44	20,488 (8.6)
						45–49	18,912 (8.9)
**Climatic region** (state abbreviations)						50–54	17,486 (8.7)
Northeast (CT, DE, ME, MD, MA, NH, NJ, NY, PA, RI, VT)			33,785 (17.1)			55–59	16,357 (8.0)
Southeast (AL, FL, GA, NC, SC, VA, DC)			22,708 (10.7)			60–64	14,410 (6.8)
Central (IL, IN, KY, MO, OH, TN, WV)			39,692 (20.2)			65–69	12,521 (5.3)
South (AR, KS, LA, MS, OK, TX)			8,771 (4.2)			70–74	9,522 (4.1)
North Central (IA, MI, MN, WI, MT, NE, ND, SD, WY)			26,768 (13.3)			75 +	17,101 (7.4)
West (CA, NV)			35,721 (17.5)				
Southwest (AZ, CO, NM, UT)			11,035 (5.2)				
Northwest (ID, OR, WA)			21,656 (11.6)				

**Fig. 1:**
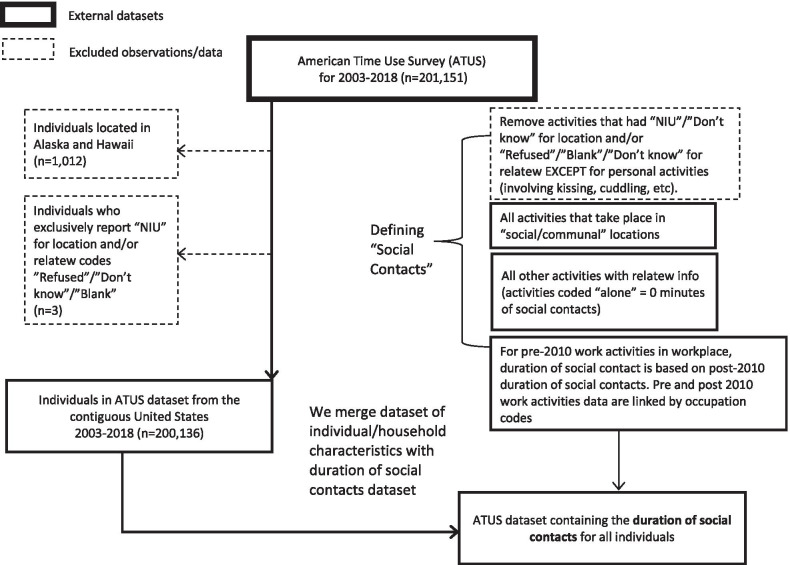
Flowchart illustrating our sample selection and how we define social contacts

Our sample consists of data from 200,136 individuals from 2003 to 2018 available in IPUMS Time Use [22]. The sample consists of 112,286 females (51.6% after weighting) and 87,850 males (48.4%) who reside in the contiguous 48 US states. For every individual, we have information on their age, sex, race, marital status, state of residence, education level, labor force status, and occupation (see Table 1 for more details). The time diaries include information on the nature and location of activities, as well as information on whom the respondent is conducting the activity with (if anyone). Information detailing the location and presence of others in the room was collected for most activities except for sleeping, grooming, and other personal activities. Respondents who exclusively reported activities that did not include information on who else was present or the location of the activity or those who refused to respond were also excluded from our final dataset (see Fig. 1). There is no information on the age of individuals the respondents have contact with when they are not household members. Moreover, the ATUS has information on adults spending time with children but lacks information on the time children under the age of 15 spend on activities alone, that involve adults, or with other children.

### Methods

All of the analyses were conducted using Stata/SE version 16 [41].

We are interested in identifying social contacts that can influence the transmission of respiratory pathogens; thus, we define a social contact as an activity done with others or in an indoor setting where others are always present. This is similar to the definition used by Zagheni et al. [46]. As detailed in the sections below, we establish rules for defining social contacts first by location, then activity type (work versus non-work), and then based on information on with whom the activity was done. We are interested in the age-pattern of social contacts, so all the findings are presented by five-year age groups.

#### Defining social contacts in the home

In the case of social contacts in the home, we consider any activity done in the respondent’s home or yard with someone else a social contact. We exclude activities for which information on location and who was present were not collected from the respondents. Similarly, we drop those activities where questions about who was present were asked but the respondent refused to answer. Sleeping and other personal activities in the home are not included because they do not have information on other people (if any) with whom the activity was done.

#### Defining social contacts outside the home

We assume all activities involved social contact for the following locations: someone else’s home; restaurant or bar; place of worship; grocery store; other store, mall; school; library; bank; gym/health club; post office; bus; subway, train; taxi, limousine service; and airplane. Most of these are public locations that typically require the presence of staff such as transportation workers, servers, or cashiers, and often include other patrons, so we assume that someone else will always be present. Moreover, these locations are all indoor settings where social contact is more likely to result in the transmission of respiratory pathogens. For the rest of the locations (except for work activities in the respondent’s workplace pre-2010 and personal activities) we assume a social contact is present only if another person is recorded as present (under the variable *relatew*) during the activity. We exclude activities for which location and who was present were not collected from the respondents, except for personal activities described as kissing, cuddling, etc. In those instances, we assume social contact is always present. Similarly, we drop those activities where questions about who was present was asked but the respondent refused to answer. See Fig. 1 for more details on exclusions from the analytic sample.

Prior to 2010, work-related social contacts (e.g., boss or manager; people whom the respondent supervises; co-workers; customers) were not recorded in the ATUS. To address this omission, we calculate the percent of time spent in social contact by detailed occupation (occ) categories for post-2010 work activities in the workplace (see Additional file 1: Figure S1 and Additional file 2: Table S1 for more information). We use that calculation to impute the percent of time spent in social contact for pre-2010 work activities in the workplace. We multiply the duration of work activities pre-2010 by the percent of work time spent in social contact for that person’s occupation category post-2010 to get an imputed duration of time spent in social contact at work pre-2010. For those detailed occupation codes that did not match between pre- and post- 2010 work activities, we either substitute post-2010 occupation codes – by using a cross-walk where available – or use the percentages from the broader occupation categories (occ2). We do not do this imputation for work activities in locations other than the workplace, post-2010 work activities in the workplace, and all non-work activities in the workplace; they are treated the same as other activities in those locations (e.g., a work activity in a restaurant or bar would be treated as always involving social contact). Finally, we sum the duration of activities with social contact for each individual using the rules described above and merge this dataset with individual and household characteristics for our analyses.

### Analysis

We plot the distribution and estimate the overall mean and standard deviation of the number and duration of household member contacts. We also plot the distribution and estimate the mean and standard deviation of the duration of all social contacts.

We stratify the sample by respondents’ demographic (gender and race/ethnicity), temporal (type of day and seasons), and spatial (climatic regions and metro/non-metro counties) characteristics. We are interested in age-patterns of social contacts, so we group respondents into five-year age groups ranging from 15-19 to 70-74, with all respondents age 75 and over in a single group.

#### Estimating the means

For each five-year age group, we find *n*_*j*_ the **mean number** and *d*_*j*_ the **mean duration**_*j*_ (in minutes) of household member contacts in the home/yard. We have age information for respondents’ household members and use that to create two different **age contact matrices (mij, who spends time with whom by age)** showing the mean number (*n*_*ji*_) and mean duration (*d*_*ji*_) of contacts for respondents in age group *j* and household member contacts in age group *i*. We use 10-year age groups for the respondents (15–24, …, 65–74, 75 +). Since we have age information for the respondents’ household members, we also group the respondents’ household members into the following age groups (0–5, 6–14, 15–24,…, 65–74, 75 +).

We also estimate the age pattern of *n*_*j*_ (the **mean number**) and *d*_*j*_ (the **mean duration,** in minutes) for all social contacts summed across all locations, as well as separately by location (respondent’s home/yard, work, public locations, and other (not shown in figures)). For locations outside of the home/yard we are not able to generate age contact matrices directly from the data. Controls are not used because the purpose of this analysis is descriptive, and only sampling weights are necessary to make the analysis representative of the target population—the civilian, non-institutionalized US population age 15 or older within the contiguous 48 states [40].

In addition to presenting means, we present standard deviations and 95 percent confidence intervals for the mean for each age-group in the Additional file 3: Dataset 1 and Additional file 4: Dataset 2.

#### Uncertainty analysis

**Fig. 2:**
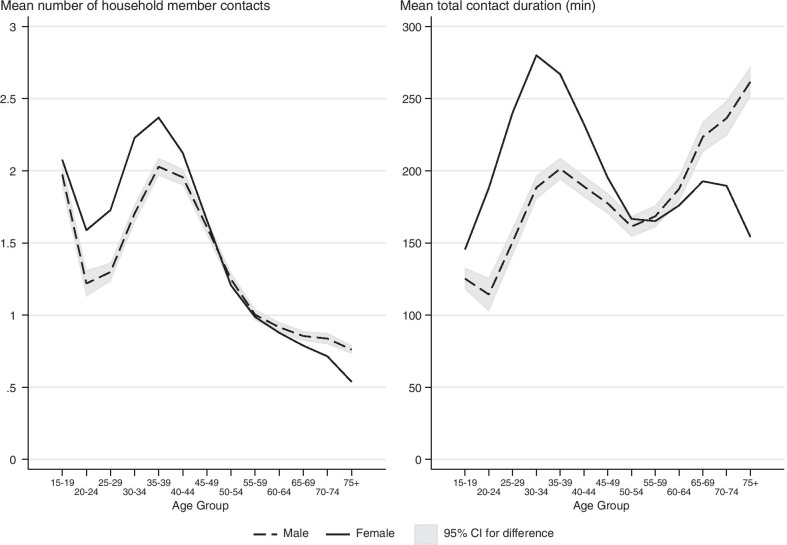
Sex and age pattern of mean number and duration of contacts with household members. The male and female contact patterns are statistically significantly different at the 95% confidence level for all age groups where the two lines do not cross the confidence intervals. The age patterns differ when we compare the mean number of contacts and the mean duration of contacts. For instance, while elderly males have the lowest mean number of household contacts among all males, they spend the most time with other household members on average. Sleeping and other personal activities are not included

We are interested in determining whether the mean number and duration of contacts (*n*_*j*_ and *d*_*j*_) differ by respondent characteristics. Therefore, in addition to presenting the estimates of the means in the figures, we also include a measure of uncertainty. For instance, to show the uncertainty associated with the age-specific contact patterns in Fig. 2, we compute the 95% confidence intervals for the difference between the male and female contact patterns for each age. We then attach that confidence interval to the curve for men. This shows which differences between the two groups are statistically significant: whenever the female estimate is outside of the confidence interval, the male and female contact patterns are significantly different from one another for that age group.

In instances where we have more than two groups, we add the confidence interval for the age-specific differences between two of the groups we wish to highlight (for instance we show the black-white difference but not the white-other difference). We do not show the confidence intervals for the other differences to reduce clutter and keep the visual display clearer. In the text we state whether the age-specific patterns between key groups are statistically different.

#### O*NET occupational analysis

We merge the ATUS data (from years 2010–2018) with data from the Occupational Information Network (O*NET), to see if there are racial differences in the types of social contacts at work by occupation (Additional file 5: Text S1 contains more detailed information on how the two datasets were merged). This subsample contains 31,069 observations (Non-Hispanic White = 20,881; Non-Hispanic Black = 3,828; Non-Hispanic Other = 1,900; and Hispanic = 4,460). The O*NET data contains a “physical proximity” variable that is an average of sampled workers’ responses within an occupation regarding the level of physical proximity experienced in the workplace. The responses are based on a five-point Likert scale, ranging from 1 – “I don’t work near other people (beyond 100 ft)” to 5 – “Very close (near touching)”. O*NET rescales the five-point Likert scale into a score ranging from 0–100. We test whether the mean O*NET scores are statistically different by workers’ racial and ethnic composition. We recode the physical proximity scores into four categories (with equal ranges) including high (e.g., dental hygienists), mid-to-high (e.g., cooks), mid-to-low (e.g. engineers), and low levels of physical proximity (e.g., loggers). We then analyze the physical proximity categories in two ways. We compare the distribution of O*NET classification categories within each race/ethnic group. For each classification category, we then break down the racial and ethnic composition of workers. Finally, we test whether the mean proximity scores differ by race and if people of different races are equally likely to work in the highest (75–100) physical proximity jobs.

## Results and discussion

On average, respondents in our sample have 1.45 (SD = 1.38) social contacts with household members daily. The mean duration of household member contacts is 189 minutes (SD = 208); many respondents report no household member contacts. The mean duration of social contacts at work is 139 minutes (SD = 216). The mean duration of contacts in public locations is 121 minutes (SD = 168). The mean duration of all social contacts is 556 minutes (SD = 262). The distribution is skewed; there is a large number of respondents who report zero social contacts (see the histogram in Additional file 6: Figures S2A, B). This skewed distribution is mirrored for all of the subgroups in the data: it holds for men and women, and for all four racial categories. It also holds separately for each age group but is more pronounced at higher ages: the standard deviation of the duration of total social contact tends to increase with age. To simplify the presentation of the comparisons across demographic groups, time periods, and geographic regions, we focus the remainder of our analysis on the mean number and duration of contacts.

### General age patterns and gender differences

#### Contacts with household members in the home

The mean number of household member social contacts in the home dips in the twenties and peaks in the thirties (Fig. 2). The mean duration of contacts also peaks in the thirties, but the dip in the twenties is limited to men and is smaller in size. This aligns with patterns of leaving the parental home followed by marriage, as the average age of first marriage in the US is 28. People in their twenties are more likely to be single and thus live alone or have few contacts with roommates. As they enter their thirties and grow their families, they tend to have a greater number of household member contacts and spend more time with children [9]. Interestingly, while elderly men (75 +) have the lowest mean number of household contacts when compared to men of other age groups, they spend the second highest mean duration of time with other household members, second only to women in their thirties. While the duration of contacts increases between the ages of 55–65 for elderly women, unlike elderly men, the time they spend with others declines again after age 70. This decline is likely because a higher proportion of elderly women than men are widowed and living alone because of lower male life expectancy. Women have higher durations of household social contact before age 50, but after that age, men spend increasingly more time with others at home, which is consistent with Glauber [15]. This gender difference is likely a reflection of gendered patterns of working outside the home and childcare when children are young.

The age patterns shown here are very similar to recently published age patterns for the United Kingdom [29]. Specifically, both the US and UK data document a peak in the mean number of contacts for young and middle-aged adults (35-39 for the ATUS data versus 40-44 for UK adults). This discrepancy in the timing of the middle age adult peak may reflect the fact that our figures are based on data from the past 15 years, whereas Klepac et al. use data from 2017-2018. We may find similar results if we restrict our sample to more recent years. On average, the mean numbers of household contacts in our ATUS-based estimates are smaller than the UK based estimates. It is possible this finding may be explained by differences in household composition or other factors, but further analysis of both datasets would be necessary to assess this possibility.

#### Duration of social contacts by location

**Fig. 3:**
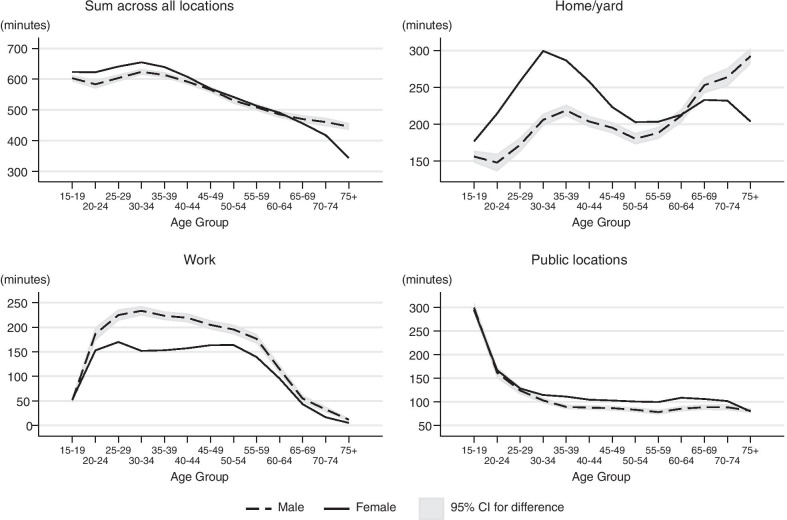
Sex and age pattern of duration of social contact by location. Social contact patterns by gender and age are related to the location of the interaction. When summing across all locations, younger women had a slightly higher duration of contact than men of the same age, while elderly men had a higher duration of contact than elderly women. However, patterns of social interaction in the home/yard and at work show important gender differences. Women have greater durations of social contact in the home or yard under the age of 55, but then men have more social interaction after age 55. In contrast, men of nearly all ages have greater duration of social contact at work but it is most evident from ages 25 to 60. In these figures, the duration of contacts at is not restricted to interactions with household members

The age pattern of total duration of social contacts (across all locations) is similar to the age pattern of the duration of household contacts: women younger than 45 have a higher duration of contact than men, while men over 65 have a higher duration of contact than women. Duration of social contact is closely aligned for men and women ages 45–64 and steadily decreases through middle age. The impact of labor force participation and family demands by gender for each location of social contacts is evident in the other panels of Fig. 3. Women under the age of 60 have a greater duration of social contact in the home/yard compared to men, while men’s duration of social contact in the home/yard increases after age 50 and surpasses women after age 60. Presumably, the greater duration of social contact at home for women under the age of 60 and the peak at age 30 is due to increased childrearing and childcare responsibilities. In contrast, men’s duration of social contact at work is higher through most of the life course. Men’s peak in mean duration of social contact at work occurs in the 30–34 age group but stays high until age 55. Women’s duration of social contact at work rises until age 25, falls during the childrearing years, and rises again until age 50. This makes sense as childrearing responsibilities typically are reduced or absent in middle age, so men and women would have more similar work and household contact patterns. Duration of social contact in public locations is at its highest for those 15–19 (because of contacts at school) and declines steeply until age 30. From age 30 to age 70, the duration of social contact in public locations is relatively stable though women have higher durations than men throughout this period.

If the duration of social contacts is strongly correlated with the number of contacts in the US as in other settings [32], then although individuals below the age of 50 are at lower risk of COVID-19 mortality than the elderly, they may be responsible for the majority of the spread through interpersonal contacts.

### Racial/ethnic differences

#### Contacts with household members in the home

**Fig. 4:**
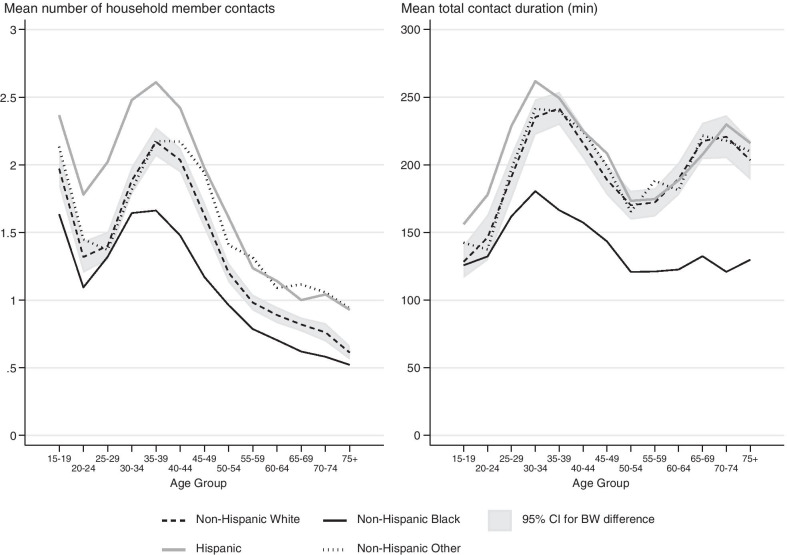
Differences in household member contact patterns by race. The confidence interval represents the difference in the confidence intervals for Non-Hispanic Black and Non-Hispanic White respondents. The Non-Hispanic Black and Non-Hispanic White age patterns are statistically different for age groups where the Black line does not cross the confidence intervals. For almost every age group, Non-Hispanic Blacks had the lowest number and shortest duration of household contacts. Hispanics under the age of 45 had the highest number of household member contacts, and those below 35 spent the most time with household members. Non-Hispanic Others and Non-Hispanic Whites had similar patterns of contacts

There are large differences in the age patterns of social contacts across different racial/ethnic groups in the United States. At home, Non-Hispanic Blacks have the lowest number and shortest duration of household member contacts compared to other race/ethnicity groups at nearly all age groups (except for 30–34 for mean number and under 25 for mean duration). Hispanics on average have the highest number of contacts below age 45, and higher durations of contacts than other groups below age 35 (Fig. 4). Non-Hispanic Others and Non-Hispanic Whites have similar numbers of contacts that are not statistically significantly different under age 39, but at older ages the mean number of contacts for Non-Hispanic Others was significantly different from Non-Hispanic Whites and similar to those of Hispanics (except for those 50–54). Non-Hispanic Whites and Non-Hispanic Others have a significantly shorter duration of social contacts than Hispanics at younger ages, but track the time-use pattern of Hispanics closely starting at age 35.

#### Duration of social contacts by location

**Fig. 5:**
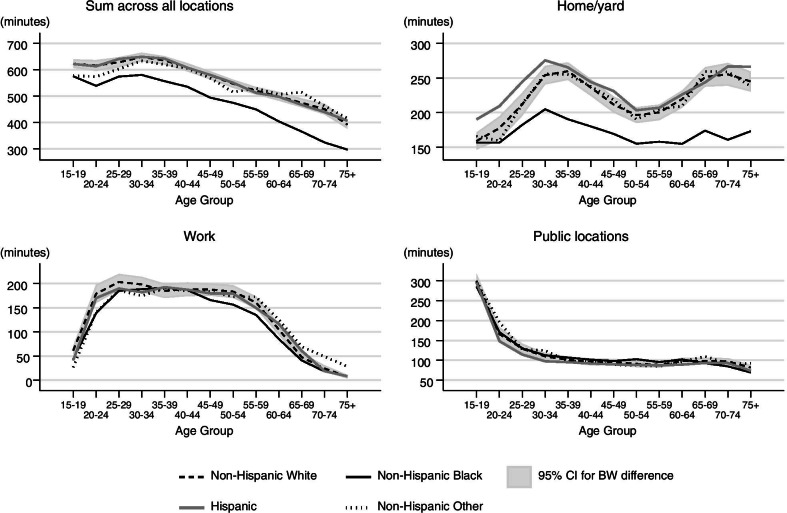
Race and age patterns of social contacts by location. The gray region represents the confidence interval for the difference between the age-specific means for Non-Hispanic Black and Non-Hispanic White respondents. The Non-Hispanic Black and Non-Hispanic White age patterns are statistically different for age groups where the Black line does not cross the confidence intervals. Non-Hispanic Blacks had the shortest duration of contact, with a substantial and persistent gap between them and Non-Hispanic Whites/Hispanics at all age groups. The latter is driven by differences in duration of contacts in the home. In this figure duration of contacts in the home/yard is not restricted to interactions with household members

We find more-nuanced patterns when we sum the mean duration of social contacts across all locations and separately for work, home/yard, and in public locations (Fig. 5). Non-Hispanic Blacks report substantially shorter durations of social contact than other racial/ethnic groups. This pattern is evident when summing across all locations and in the home/yard for those 20 or older. At work, Non-Hispanic Blacks have shorter durations of social contacts for ages 20–25 and again for ages 45–60 compared to Non-Hispanic Whites and Hispanics. Hispanics and Non-Hispanic Whites have nearly identical age patterns of durations of social contact when summing across all locations and at work. In contrast, in the home/yard, Hispanics under the age of 30 have a greater duration of social contact compared to Non-Hispanic Whites. In public locations, patterns of the duration of social contacts does not vary by race/ethnicity groups in these data.

Our findings indicate that Non-Hispanic Blacks have fewer contacts and shorter durations of contacts compared to other groups. Though these results are based on data from a period of time before the COVID-19 pandemic and stay-at-home orders were issued, these results were unexpected because Non-Hispanic Blacks appear to have a higher risk of contracting and dying from COVID-19 [5, 19]. One might expect this disparity to be partially explained by higher number of contacts or higher duration of social contacts. However, we reason that if the number and duration of social contacts during the pandemic remain similar to how they were beforehand, these differences are not likely responsible for Black-White racial disparities in COVID-19 infections and deaths.

#### Results for O*NET occupational analysis

**Fig. 6:**
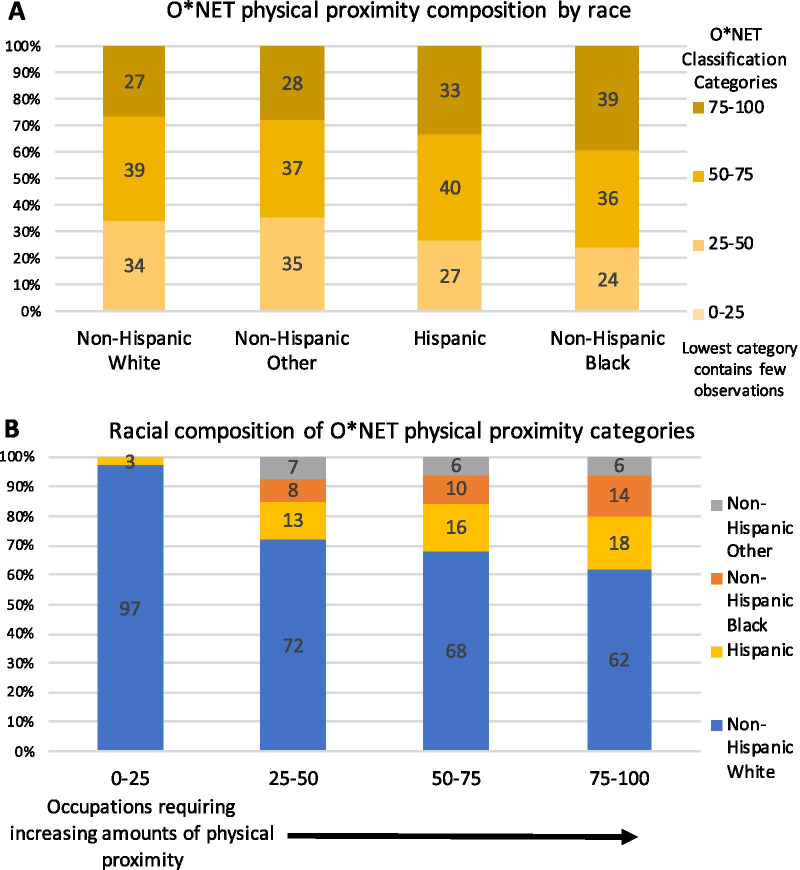
Racial differences in O*NET occupational physical proximity. **(A)** O*NET physical proximity category composition by race shows that Non-Hispanic Blacks have the highest proportion of jobs with the highest level of physical proximity. **(B)** Racial composition of O*NET physical proximity categories show that Non-Hispanic Whites make up a decreasing share and Non-Hispanic Blacks make up a correspondingly increasing share of the highest physical proximity categories

Considering these results, we combine O*NET data regarding social contact patterns by occupation with our nationally representative sample. The additional analysis reveals that Non-Hispanic Blacks are more likely to work in occupations with higher levels of physical proximity which increases disease risk (Fig. 6) (see Additional file 5: Text S1 for more details on this analysis). Figure 6A shows that Non-Hispanic Blacks have the highest proportion (39%; 12 percentage points higher than Non-Hispanic Whites p < 0.001) of occupations with high levels of physical proximity and the lowest proportion of occupations with mid-to-low levels of physical proximity. The racial differences are even more prominent in panel B, where the bars displaying the racial composition of physical proximity categories show that as the physical proximity of jobs increases, Non-Hispanic Whites make up a decreasing share and Non-Hispanic Blacks make up a correspondingly increasing share of workers. The proportion of Hispanics also increases across the top three physical proximity categories, while the proportion of Non-Hispanic Others stays essentially the same. The lowest physical proximity category contains a very small number of observations, and only consists of Non-Hispanic Whites and Hispanics.

The mean O*NET physical proximity score is highest for Non-Hispanic Blacks (mean = 65, SD = 16) followed by Hispanics (mean = 64, SD = 16), which are both statistically significantly different from Non-Hispanic Whites (mean = 61, SD = 17) (p < 0.0001). Thus Non-Hispanic Blacks may on average have higher risk/intensity contacts due to the physical proximity levels associated with their occupations, even though they may have an overall lower number and duration of contacts. This may help explain the COVID-19 racial disparities in infection and mortality rates particularly if these workers are more likely to be deemed essential.

### Temporal Differences

#### Contacts with household members in the home

**Fig. 7:**
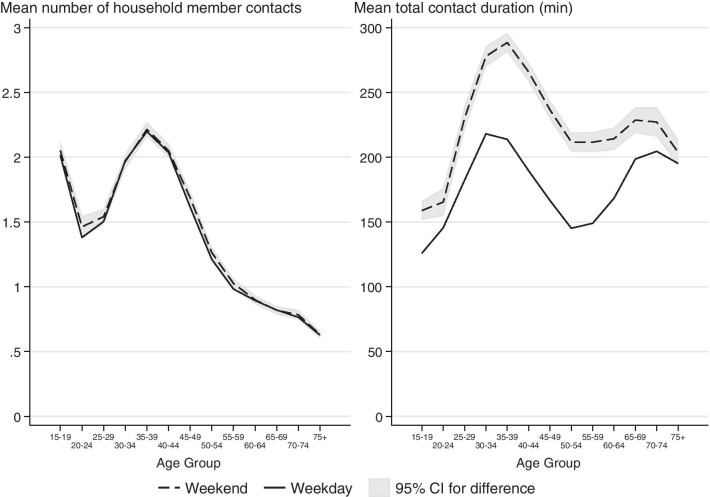
Differences in household contacts during Weekdays and Weekends. There is no statistically significant difference between the mean number of household member contacts during the weekend and weekdays. Across all but the oldest age group, the mean duration of household member contacts is longer during the weekend compared to weekdays

We investigate the temporal differences in the number and duration of household contacts by examining patterns by weekend/weekday as well as by season. When restricting our analysis to household member contacts by weekend/weekday, we do not find that the number of weekend contacts is meaningfully higher than the number of weekday contacts (Fig. 7). There is a small increase in the number of non-household member contacts in the home during the weekend (analysis not shown). However, the duration of household contacts is higher for weekends versus weekdays throughout the life course. We did not find seasonal differences in the number of household contacts (see Additional file 7: Figure S3A). However, we did find that respondents below the age of 50 spend more time with household members during the winter months.

#### Duration of social contacts by location

**Fig. 8:**
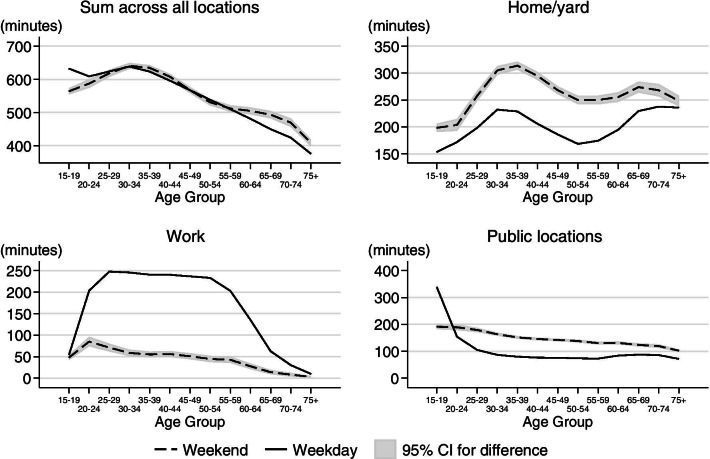
Differences in social contacts during weekdays and weekends by location. The total duration of social contact is different on weekends and weekdays for those under 20 (greater on weekdays) and over 60 (greater on weekends). However, duration of social contact is greatest in the home/yard on weekends and at work on weekdays. The duration of social contacts in public is greatest for those under 20 on weekdays, while for all other age groups social contacts in public is greatest on the weekends. In this figure duration of contacts at home/yard is not restricted to interactions with household members

The duration of total social contacts summarized across all locations was not significantly different for weekdays versus weekends for those age 25 to 60 (Fig. 8). However, the duration of social contacts was greater on weekdays for those at younger ages and greater on weekends for those at older ages. The difference in the total duration of social contacts between weekdays and weekends was statistically significant at work, in the home/yard, and in public places. Social contacts at work primarily occurred on weekdays for most of the life course. In contrast, social contacts in the home/yard and in public locations happened primarily on the weekends, the exception being that respondents 19 and younger spent more time in public locations (school) on weekdays (Fig. 8). We do not find seasonality in the duration of social contacts for any age group except for respondents below age 20 (see Additional file 7: Figure S3A). For the youngest respondents, they spend statistically significantly less time with others during the summer months; this result is driven by the school term.

These results imply that if the main mechanism driving infectious disease seasonality is seasonality in contact patterns, then we should not expect to see large seasonal differences in disease incidence when the young are not very susceptible or infectious, as may be the case for COVID-19 [47]. Disease seasonality would have to be driven by other factors such as riskiness of contacts (e.g. proximity, indoor vs. outdoor), seasonality of pathogen survival outside the host, or seasonal changes in host immunity [17].

### Spatial differences in contact patterns

We do not find many differences in contact patterns between metro and non-metro areas. The main exception is that 15–34-year-olds and those age 50 and older spend more time with others in the home/yard in non-metro areas, this difference was statistically significant (see Additional file 7: Figure S3B). This was unexpected given recent findings in the UK. The UK participants who spent time in low density locations (< 1000 people per km) recorded fewer contacts than participants who spent time in more dense locations (> 1000 people per km) [29]. Interestingly, Read et al. [37] found no differences between the number and duration of contacts between administratively defined rural and urban populations in China, but did find statistically significant differences when stratifying based on population density. In contrast to the Klepac et al. [29] finding, they find fewer contacts in the highest density locations. It is possible that we would find spatial differences in contact patterns if we used a definition more closely tied to population density. Based on data from 2015–2019 American Community Surveys, which include the same metro versus non-metro definition but also have information on population density, the mean population density in non-metro counties (mean = 15 per km, SD = 35 per km) is much lower than the mean population density of metro counties (mean = 215 per km, SD = 769 per km). Nevertheless, the range of population densities that can be found in non-metro areas is large (range = 0.0140–1107 persons per square kilometer).

The age pattern of duration of social contacts are very similar across the nine climatically consistent regions [28] (see Additional file 7: Figure S3C for more details). One region that deviates from the rest is the West Region (CA and NV); the mean duration of work contacts for adults between the ages of 25–55 was consistently shorter (but not always statistically significant) when compared to other regions (see Additional file 7: Figure S3C). These results suggest that differences in climate may not be a major driver of differences in social contact patterns. Although these regions are climatically different, differences in human behavior/culture may matter more for contact patterns. In the context of the COVID-19 pandemic, spatial differences in contact patterns will emerge in places with different control measures aimed at limiting contacts. For instance, Feehan and Mahmud [13] who study social contacts patterns across six US metro areas during the COVID-19 pandemic did not find much regional variation in contact patterns in April 2020 when most cities had lockdowns,however differences emerged over the course of the next few months as policies varied across space [13]. Because responses to COVID-19 have been politicized and political attitudes are spatially polarized, the pandemic is likely to have induced major spatial differences in contact patterns.

### Age contact matrices

**Fig. 9:**
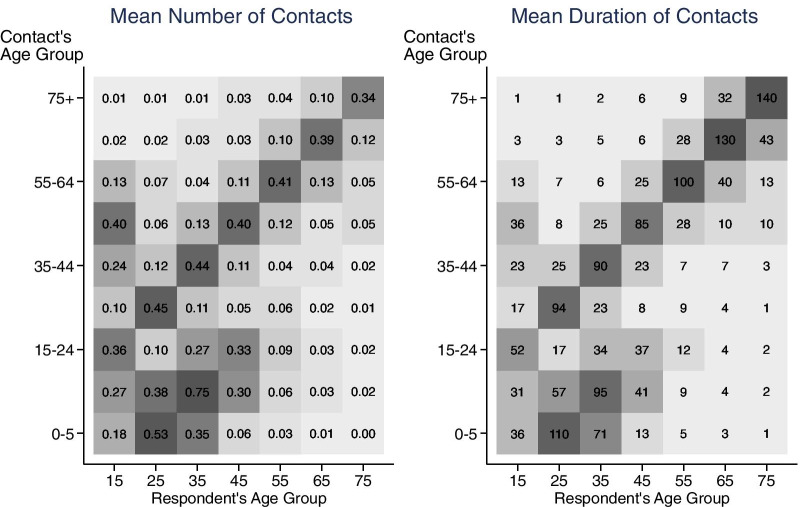
Age contact matrices showing mean number and mean duration of household member contacts. The darker the color of the matrix cell the higher the number and duration of contact. They display assortative contacts by age, and people one generation apart spending time with each other (parents and children). Based on ATUS 2003–2018 surveys. ATUS respondents had to be at least 15 years old

Recall that the ATUS only has data on the age of respondents’ contacts if the contacts are household members. Therefore, we can create age contact matrices to describe social contacts between respondents and household members. The age contact matrix showing the mean number of household contacts (Fig. 9) documents assortative contacts with age (siblings spending time with siblings, and similar aged couples or roommates spending time with each other). There is also evidence of people one generation apart spending time with each other (parents and their children). Our results share some of the same features found in the matrices from the POLYMOD and a recent UK survey [29, 34]. In contrast, our household contact matrix differs substantially from the Melegaro et al. [32] matrix for the Manicaland Province of Zimbabwe, where household sizes are larger and extended families are more common. Future work will attempt to identify which POLYMOD country the US data is most similar to.

The age-structured contact matrix showing the duration of contacts (duration of exposure matrix) is qualitatively similar to the Zagheni et al. [46] household member duration of exposure matrix (Fig. 9 and see Zagheni Fig. 3). The line graphs are consistent with the patterns in the age contact matrices, in which you can see that the total number of contacts is decreasing with respondent age, while the total duration is high for younger and older age groups but dips in the middle.

**Fig. 10:**
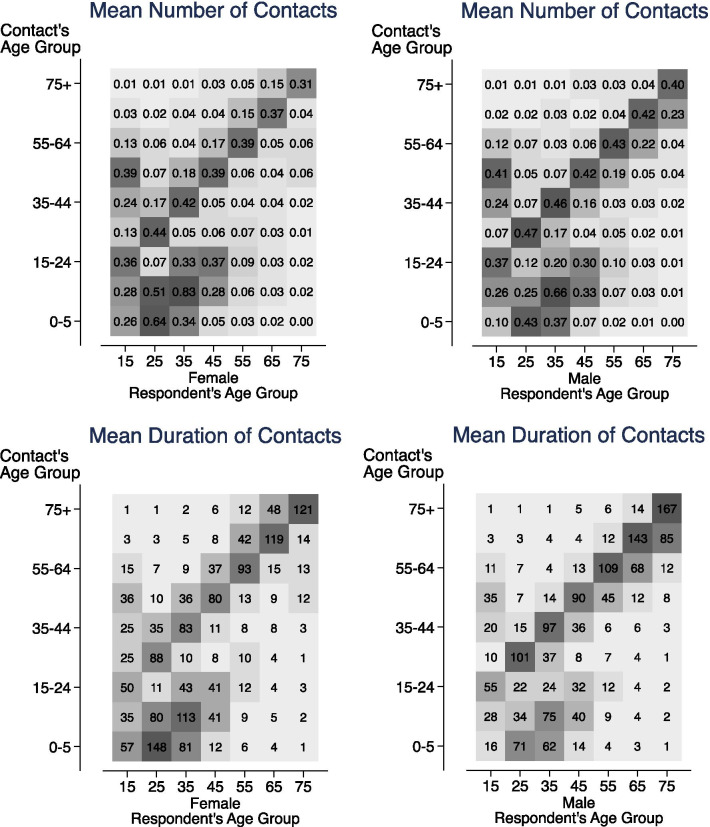
Age contact matrices showing the mean number and duration of household member contacts by sex. They display assortative contacts by age, and show parents, especially mothers, spending more time with children. Women have more contact with the age group directly above theirs, while men have more contact with the age group directly below theirs. Based on ATUS 2003–2018 surveys. ATUS respondents had to be at least 15 years old

In Fig. 10, which breaks down the age contact matrices by sex, we can see that women ages 25–44 spend much more time with children and have higher numbers of contacts with them than men. On average, women spend more time on child-rearing in dual-parent households [4], and a higher proportion of women are single parents than men; for these reasons, women would have more contacts with children. Women have higher numbers of contacts and longer durations of contact with people in the 5-year age groups directly above theirs, while men have the exact opposite pattern, with higher numbers and durations of contact in the 5-year age groups directly below theirs. This is likely explained by the age gap in married couples, where women’s partners skew older, with 45% falling between 2–9 years older. As shown in the line graphs, elderly men report higher numbers and durations of contact with their own age groups (and those directly below theirs) because they are less likely to be widowed than elderly women.

## Conclusion

Comparing demographic, temporal, and spatial patterns of social contacts, we find the greatest variation in social contacts based on demographic factors such as age, gender, and race/ethnicity. We also find some temporal differences in social contact patterns, though the largest differences are in the duration of social contacts as opposed to the number of social contacts. Prior to the pandemic, we do not document meaningful differences in social contact patterns across regions or when comparing metro and non-metro areas, despite the large geographic size of the US. However, urbanness and regional location may have more of an impact on the timing of the start of a local epidemic than on its overall spread or the speed of transmission [18]. Of particular note, these findings suggest that if urban and spatial differences in disease incidence existed prior to the pandemic, they may have been due to other factors besides social contact patterns [37]. On the other hand, the pandemic and political polarization may have induced new spatial differences in contact patterns that currently impact infectious disease transmission rates.

In addition to differences by key demographic characteristics, we also find distinct patterns when we compare the number and duration of social contacts. In particular, the household member contact results illustrate that although there are similarities, the age pattern of the number of contacts may differ from the age pattern of the duration of contacts. This is relevant because although both the number and duration of contacts matter for disease transmission, one might be more important than the other depending on the disease [37].

There are several important limitations to our results. The ATUS data do not include respondents below age 15. We only know the age and sex of the respondents’ contacts if they are household members. Therefore, we are not able to create age-specific contact matrices for non-household locations without making some assumptions about proportionate time mixing, which may not always be good approximations [46]. Also, the data do not specify the type of social contact (conversational versus physical) and the proximity of contacts (apart from the O*NET analysis). However, some information may be inferred based on the description of the activity. Additionally, ATUS does not include active military personnel and people residing in institutions such as nursing homes and prisons, while the latter may be some of the most vulnerable to infectious disease spread and impact. Moreover, social contact data for work hours does not exist before 2010. Though we do not have evidence that occupational contacts have systematically changed between the two periods, future research is needed to investigate changes in social contact patterns by occupation over time. Ideally, future studies of US social contact patterns would include children, military personnel, and people residing in institutions. For the ATUS to be more helpful, additional survey questions are needed to identify the number and ages of people a respondent is in contact with outside the household during each activity. Although this can be done, there are few surveys that combine contact and time-use data collection methods [32].

Nevertheless, the ATUS data allows us to estimate social contacts before substantial social distancing measures were implemented to control the COVID-19 pandemic. For the US this may be one of the only sources of nationally representative pre-pandemic social contact data. This data can help us analyze the effectiveness of social distancing measures by comparing the pre-pandemic social mixing patterns and matrices with changing contact patterns under different mitigation strategies. For instance, do we see changes in contacts occurring in the respondents’ house/yard, such as increased contacts with household members and neighborhood kids, or decreased contacts with grandparents who are in higher-risk age groups? We can also examine whether the total duration of contacts in places like restaurants, shopping centers, workplaces, schools, public transportation, and grocery stores has declined as a result of physical distancing measures.

Another advantage of the ATUS data is that we are able to disaggregate the social mixing data by geographic region and respondents’ sociodemographic characteristics. Though our analytic sample size is increased by pooling multiple years together, we are careful to ensure that there are sufficient observations within each category for the results to be meaningful. This disaggregation is important because the US is large and heterogeneous, and social distancing measures have not been uniformly enacted or embraced. Social determinants (e.g., socioeconomic status, metropolitan vs non-metropolitan areas, and occupation) can impact both baseline social contact patterns and the ability to physically distance. Moreover, disaggregation can identify who remains most at risk and where testing and interventions should be targeted to prevent spread.

## Supplementary Information

Below is the link to the electronic supplementary material.

**Additional file 1: Figure S1. **Average duration (in minutes) of social contact at work by occupation code 2010-2018.

**Additional file 2. Table S1. **Average Duration (in minutes) of Social Contact at Work by Occupation Code 2010-2018 Created by Audrey Dorélien (dorelien@umn.edu) based on ATUS data.

**Additional file 3. Dataset 1. **Excel file containing additional information (95% confidence intervals, standard deviation, and coefficient of variation) for age pattern figures.

**Additional file 4. Dataset 2. **Excel file containing additional information (95% confidence intervals, standard deviation) for age-structured contact matrices figures.

**Additional file 5. Text S1. **O*NET Data and Methods.

**Additional file 6. Figure S2. A **Histogram showing the distribution of duration of social contacts in minutes in the ATUS sample. **B** Histogram showing the distribution of duration of social contacts by age group in the ATUS sample.

**Additional file 7. Figure S3. A** Seasonal differences in mean duration of total social contacts by location. **B** Mean duration (minutes) of total social contacts for metro versus non-metro respondents by age groups and location. **C** Regional Differences in mean duration of total social contact by age group and location.

## Data Availability

The data used for this analysis are available after signing up from the ATUS Extract Builder database available at https://www.atusdata.org. The O*Net data can be found at the following location https://www.onetonline.org/find/descriptor/result/4.C.2.a.3. The scripts to run the analyses can be found at https://www.dropbox.com/sh/785vte5rpqwnzeg/AAAhO8dAWCOBHgybwga6B-DWa?dl=0.
