# Supplementary material for: Analyzing the demographic, spatial, and temporal factors influencing social contact patterns in U.S. and implications for infectious disease spread
Source: BMC Infect Dis. 2021 Sep 27;21:1009. doi: 10.1186/s12879-021-06610-w (PMC8474922; doi:10.1186/s12879-021-06610-w)
Supplement: Supplementary file 1 — Additional file 1: Figure S1. Average duration (in minutes) of social contact at work by occupation code 2010-2018. [file 12879_2021_6610_MOESM1_ESM.pdf]

# Figure S1: Average duration (in minutes) of social contact at work by occupation code 2010-2018

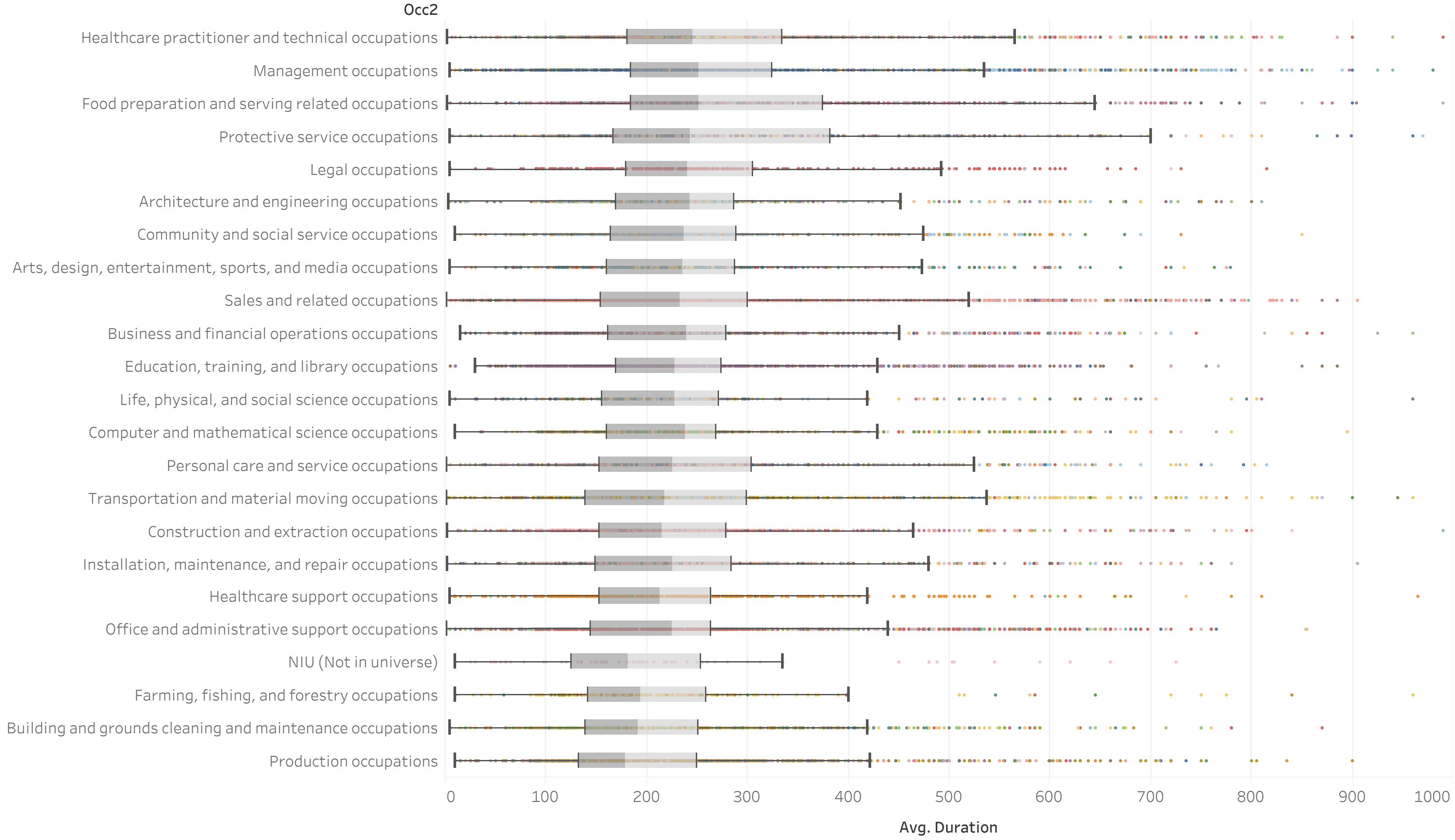


Average of Duration for each Occ2. Color shows details about Occ. Details are shown for Caseid.
