# Supplementary material for: Analyzing the demographic, spatial, and temporal factors influencing social contact patterns in U.S. and implications for infectious disease spread
Source: BMC Infect Dis. 2021 Sep 27;21:1009. doi: 10.1186/s12879-021-06610-w (PMC8474922; doi:10.1186/s12879-021-06610-w)
Supplement: Supplementary file 2 — Additional file 2. Table S1. Average Duration (in minutes) of Social Contact at Work by Occupation Code 2010-2018 Created by Audrey Dorélien (dorelien@umn.edu) based on ATUS data. [file 12879_2021_6610_MOESM2_ESM.pdf]

Table S1.

Average Duration (in minutes) of Social Contact at Work by Occupation Code 2010-2018

Created by Audrey Dorélien (dorelien@umn.edu) based on ATUS data

Occ2	
Healthcare practitioner and technical occupations	247.96
Management occupations	245.71
Food preparation and serving related occupations	245.58
Protective service occupations	242.28
Legal occupations	235.46
Architecture and engineering occupations	224.93
Community and social service occupations	224.03
Arts, design, entertainment, sports, and media occupations	221.63
Sales and related occupations	220.08
Business and financial operations occupations	216.41
Education, training, and library occupations	216.04
Life, physical, and social science occupations	212.22
Computer and mathematical science occupations	212.04
Personal care and service occupations	209.84
Transportation and material moving occupations	206.44
Construction and extraction occupations	204.60
Installation, maintenance, and repair occupations	202.80
Healthcare support occupations	201.58
Office and administrative support occupations	196.37
NIU (Not in universe)	194.63
Farming, fishing, and forestry occupations	189.51
Building and grounds cleaning and maintenance occupations	185.77
Production occupations	179.08

Average of Duration broken down by Occ2.
