# Supplementary material for: Analyzing the demographic, spatial, and temporal factors influencing social contact patterns in U.S. and implications for infectious disease spread
Source: BMC Infect Dis. 2021 Sep 27;21:1009. doi: 10.1186/s12879-021-06610-w (PMC8474922; doi:10.1186/s12879-021-06610-w)
Supplement: Supplementary file 5 — Additional file 5. Text S1. O*NET Data and Methods. [file 12879_2021_6610_MOESM5_ESM.pdf]

Text S1: O*NET Data and Methods

Description of O*NET Data:

The Occupational Information Network (O*NET) database is the nation's primary source of occupational information and is developed under the sponsorship of the U.S. Department of Labor/Employment and Training Administration. The O*NET database contains hundreds of standardized and occupation-specific descriptors across six domains on 974 occupations covering the entire range of the U.S. economy. The O*NET-SOC taxonomy is based on the Standard Occupational Classification (SOC) and defines the set of occupations across the world of work. The O*NET database is free and publicly available and is continually updated from input by a broad range of workers in each occupation ("About O*NET," n.d.).

Data collection began in 2001 and incorporates a two-stage design in which first, a statistically random sample of businesses expected to employ workers in the targeted occupations are identified, and second, a random sample of workers in those occupations within those businesses are selected. New data is collected by surveying job incumbents using standardized questionnaires ("O*NET Data Collection Overview," n.d.). Physical proximity is collected under the domain of occupational requirements and work context and asks respondents to answer the question "How physically close to other people are you when you perform your current job?" on a 5-point scale of increasing physical proximity, from 1 - "I don't work near other people (beyond 100 ft)" to 5 – "Very close (near touching)" ("Work Context: Physical Proximity,", n.d.).

Creating crosswalk:

To integrate the O*NET physical proximity data with ATUS race and occupation, we used a O*NET/Census and 2010 Standard Occupational Classification crosswalk generated by Jennifer Hook. O*NET occupation codes are a more detailed variant of the 2010 Standard Occupational Classification (SOC) codes. O*NET's SOC scheme consists of 974 occupation codes, while SOC 2010 uses only 749 codes. There is no prevalence data for O*NET's more detailed occupation codes. Eighty-five percent of SOC codes link directly to one O*NET code. For the rest, the majority of the variation (57-85%) is found to be between SOC codes and not within detailed sub-occupations within SOC codes. Therefore, the first O*NET match was selected to trim O*NET to 749 SOC codes, taking the first value and dropping the other detailed variants.

Then the crosswalk was generated by merging the Census occ codes and the 2010 SOC codes. This created 882 records (matched to allow cases from each dataset). The file was then manually edited to reduce the crosswalk to Census codes. Although many finer detailed SOC codes were deleted, some SOC/O*NET codes had to be applied to multiple Census codes (e.g., the Census codes are more detailed for types of nurses). The SOC/O*NET codes that most closely matched the Census description were selected, with a preference to codes that had O*NET data (there were many "remainder" categories - i.e. "all other" occupation categories that did not have O*NET data).

Merging crosswalk with O*NET and ATUS data:

The crosswalk was then merged with the O*NET physical proximity dataset, resulting in 514 merged records. The resultant dataset was merged with an ATUS dataset containing

respondents with work activities and sociodemographic information (including race and occupation) for years 2010-2018, excluding Alaska and Hawaii. Ninety five percent (31,069 records) matched. The majority (72%) of ATUS records that didn't match were NIU (Not in Universe) for occupation. The remaining unmatched ATUS records were distributed across only a handful of occ codes, which were in "all other" or "miscellaneous" categories. Physical proximity was then collapsed and tabulated by respondent race to obtain mean estimates and percentages.

References

"About O*NET." *O*NET Resource Center*, National Center for O*NET Development, www.onetcenter.org/overview.html. Accessed 15 July 2020.

"O*NET® Data Collection Overview." *O*NET Resource Center*, National Center for O*NET Development, www.onetcenter.org/dataCollection.html. Accessed 15 July 2020.

"Work Context: Physical Proximity." *O*NET OnLine*, National Center for O*NET Development, www.onetonline.org/find/descriptor/result/4.C.2.a.3. Accessed 15 July 2020.
