# Supplementary material for: Analyzing the demographic, spatial, and temporal factors influencing social contact patterns in U.S. and implications for infectious disease spread
Source: BMC Infect Dis. 2021 Sep 27;21:1009. doi: 10.1186/s12879-021-06610-w (PMC8474922; doi:10.1186/s12879-021-06610-w)

Figure S2A. Histogram showing the distribution of duration of social contacts in minutes in the ATUS sample.

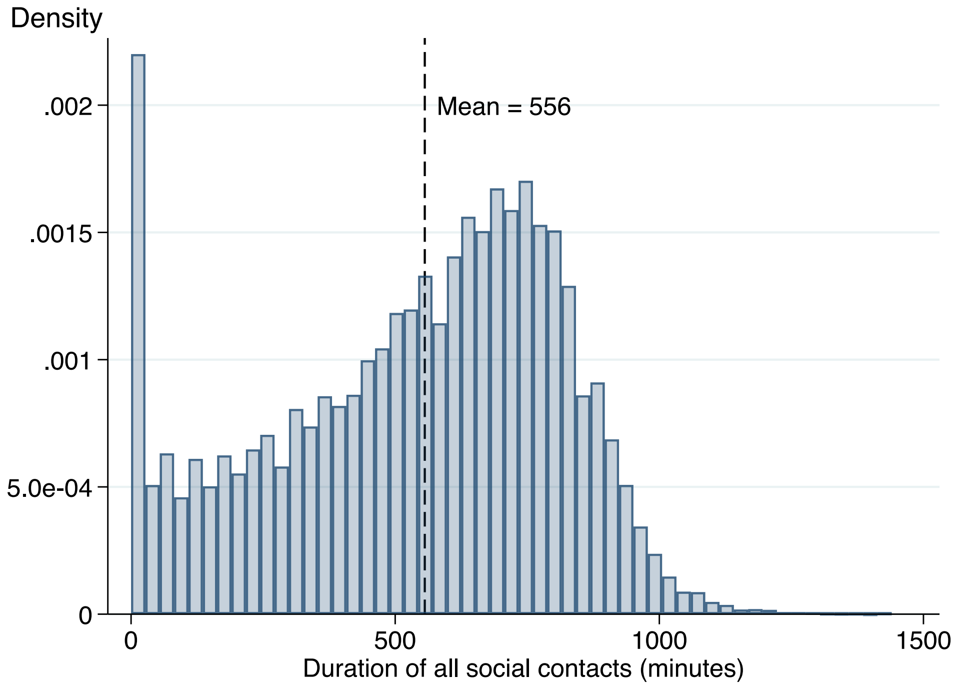

Figure S2B. Histogram showing the distribution of duration of social contacts by age group in the ATUS sample.

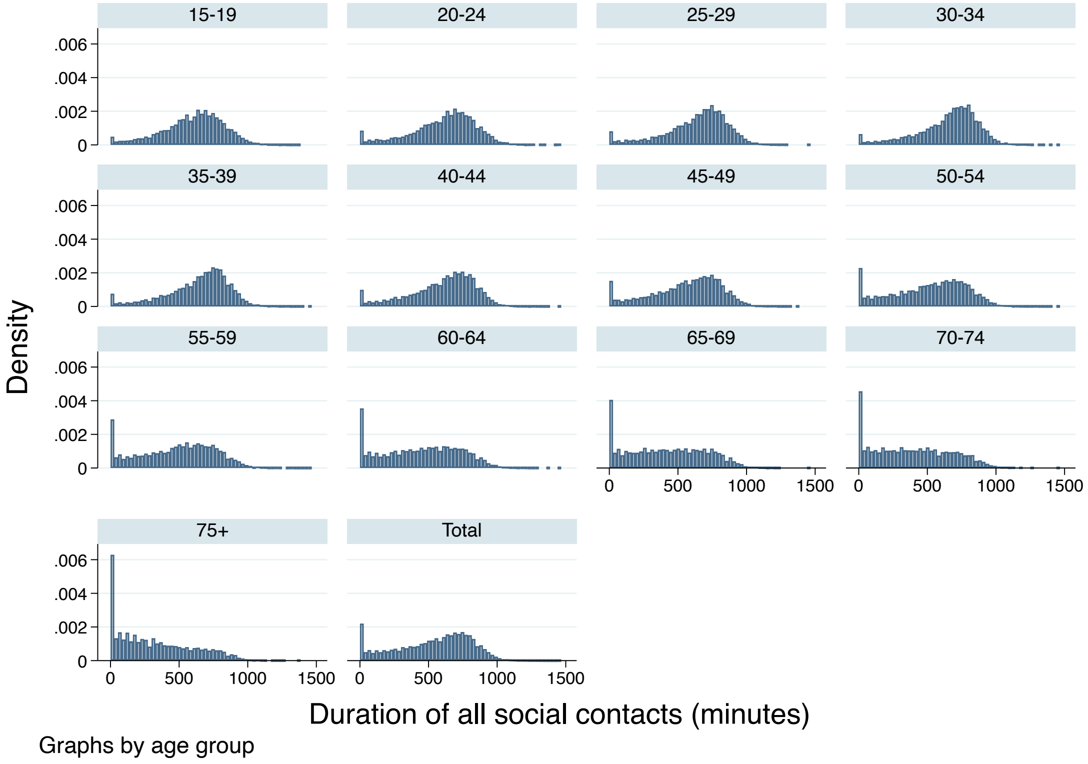

Supplement: Supplementary file 6 — Additional file 6. Figure S2. A Histogram showing the distribution of duration of social contacts in minutes in the ATUS sample. B Histogram showing the distribution of duration of social contacts by age group in the ATUS sample. [file 12879_2021_6610_MOESM6_ESM.pdf]
