# Supplementary material for: Analyzing the demographic, spatial, and temporal factors influencing social contact patterns in U.S. and implications for infectious disease spread
Source: BMC Infect Dis. 2021 Sep 27;21:1009. doi: 10.1186/s12879-021-06610-w (PMC8474922; doi:10.1186/s12879-021-06610-w)

Figure S3A. Seasonal differences in mean duration of total social contacts by location.

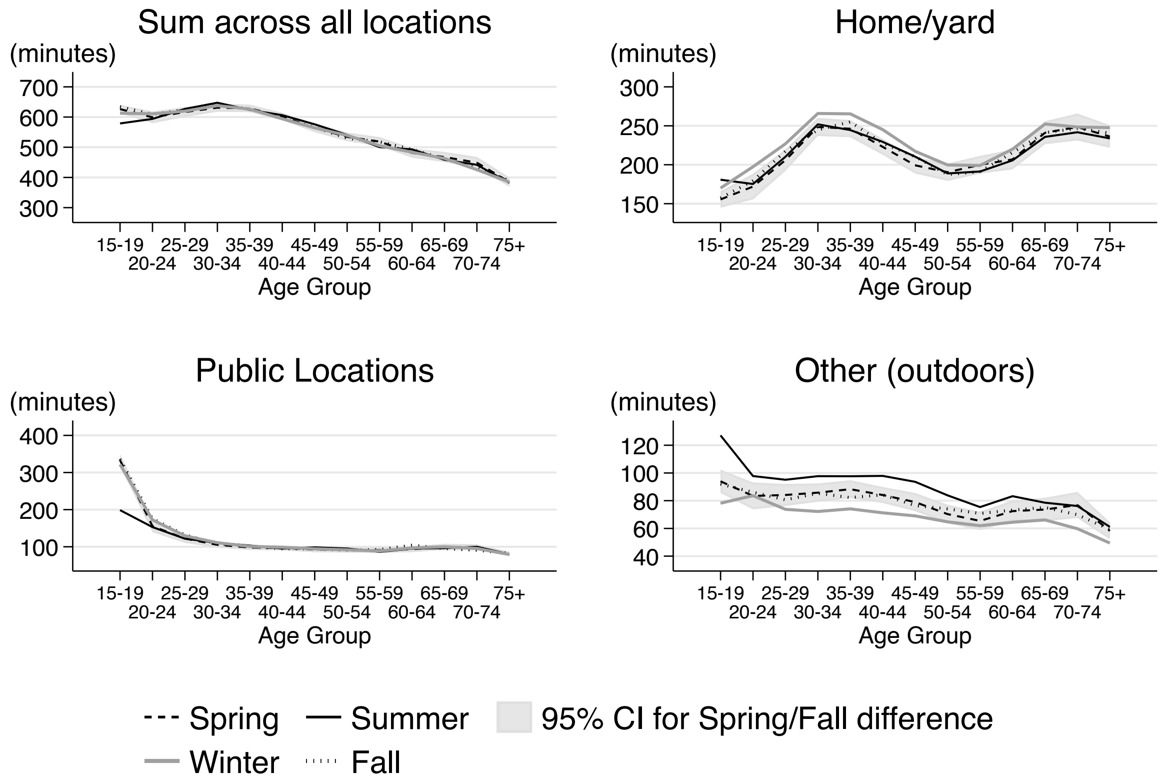

Note: Duration of work contacts did not vary by season

Figure S3B. Mean duration (minutes) of total social contacts for metro versus non-metro respondents by age groups and location.

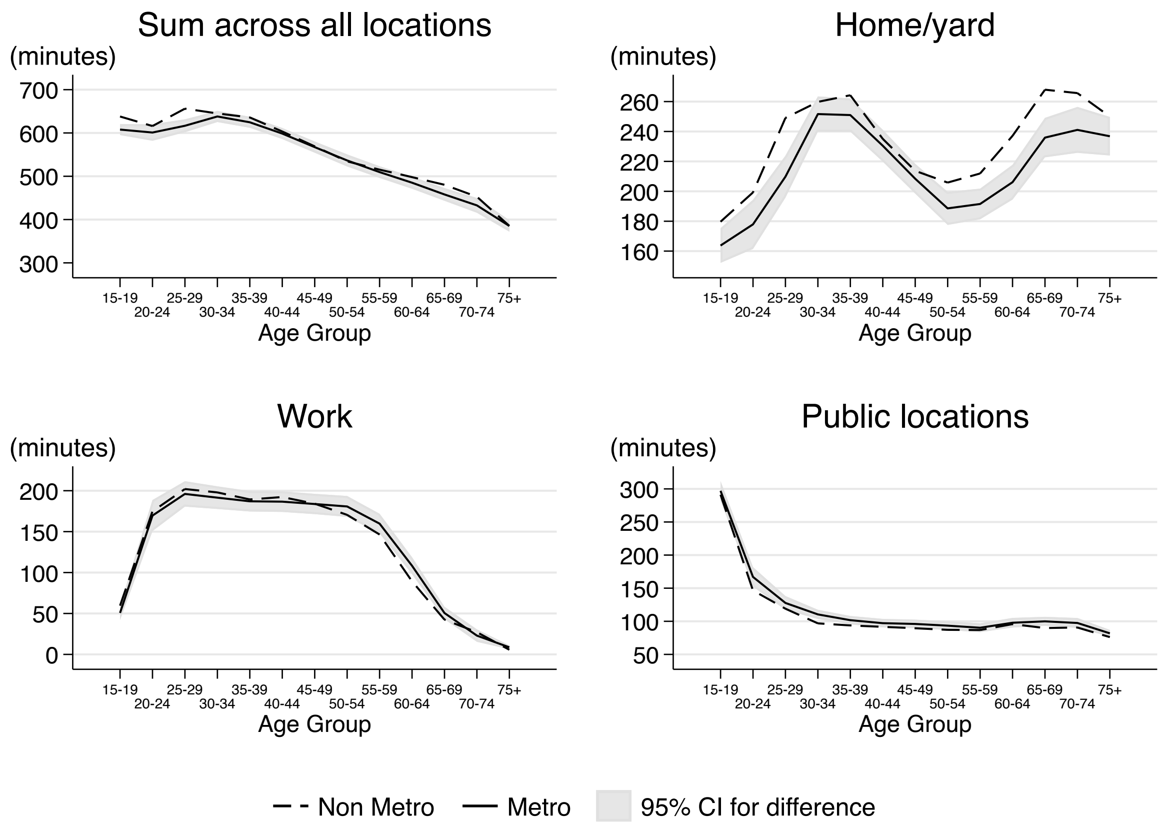

Figure S3C. Regional Differences in mean duration of total social contact by age group and location.

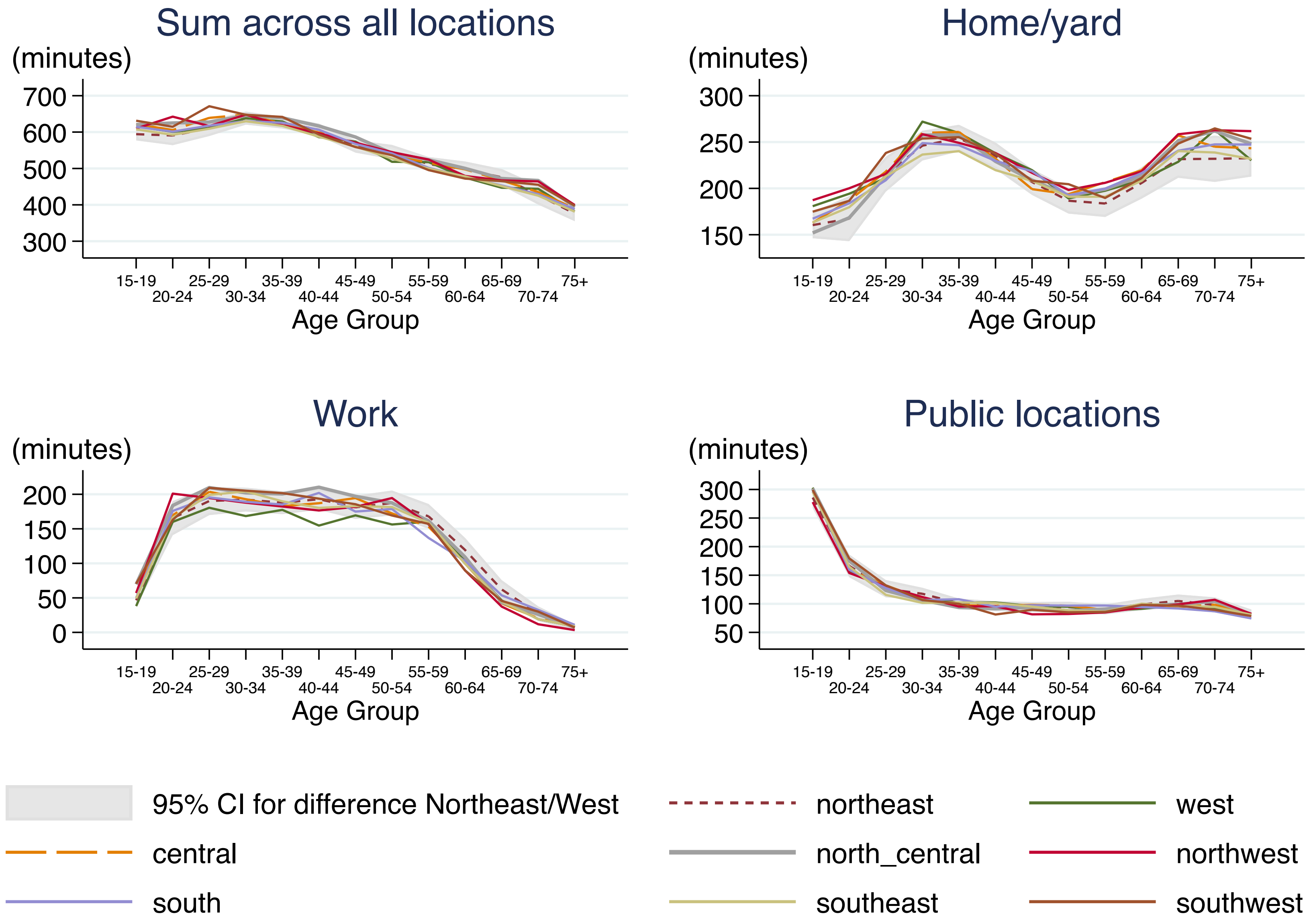

Supplement: Supplementary file 7 — Additional file 7. Figure S3. A Seasonal differences in mean duration of total social contacts by location. B Mean duration (minutes) of total social contacts for metro versus non-metro respondents by age groups and location. C Regional Differences in mean duration of total social contact by age group and location. [file 12879_2021_6610_MOESM7_ESM.pdf]
